# Post-traumatic epidural tension pneumocephalus: a case report

**DOI:** 10.1186/s13256-015-0633-5

**Published:** 2015-06-26

**Authors:** Vidar Rao, Oddrun Fredriksli, Sasha Gulati

**Affiliations:** 1 Department of Neurosurgery, St. Olavs Hospital, Trondheim, Norway; 2 Department of Neuroscience, Faculty of Medicine, Norwegian University of Science and Technology, Trondheim, Norway

**Keywords:** Computed tomography, Tension pneumocephalus, Trauma

## Abstract

**Introduction:**

Pneumocephalus is usually a self-limiting condition commonly associated with neurosurgical interventions, head and facial trauma. In contrast, tension pneumocephalus is extremely rare, and considered a neurosurgical emergency.

**Case presentation:**

We present a rare case of post-traumatic epidural tension pneumocephalus in a 30-year-old white man who deteriorated rapidly after a blunt head trauma. Imaging revealed a large, right temporoparietal epidural pneumocephalus with mass effect, most likely arising from a small defect in the mastoid sinus. A pre-existing mucocele was also suspected. Emergency burr hole evacuation was performed and he experienced full recovery, but more invasive treatment was eventually needed to resolve the condition.

**Conclusions:**

Epidural tension pneumocephalus is a rare and potentially life-threatening condition, but treatable with the right management. To the best of our knowledge, a post-traumatic tension pneumocephalus caused by a pre-existing mucocele has not been reported in the literature.

## Introduction

Intracranial air entrapment (pneumocephalus) is commonly seen after head and facial trauma, ear infections, tumours of the skull base or neurosurgical interventions [1]. It has also been reported to occur spontaneously, but this is considered extremely rare, with less than a dozen reported cases in the literature [2]. In tension pneumocephalus the continuous accumulation of intracranial air is thought to be caused by a “ball valve” mechanism [3]. In turn, this may lead to a mass effect on the brain, with subsequent neurological deterioration and signs of herniation. Tension pneumocephalus is considered a life-threatening, neurosurgical emergency.

We report a rare case of post-traumatic epidural tension pneumocephalus in a man who deteriorated rapidly after a blunt head trauma. Imaging revealed a large, right temporoparietal epidural pneumocephalus with mass effect and midline shift, most likely arising from a small defect in the mastoid sinus. Burr hole evacuation was performed and he experienced full recovery. However, more invasive surgery was needed to resolve the condition.

## Case presentation

A 30-year-old white man was admitted to his local hospital after he was found unconscious in his home. His past medical history included a history of drug abuse, bipolar disorder and chronic hepatitis C. He had no history of headaches. The family reported that he was last seen when he returned home the same morning about 5 hours prior to admission. Before he went to bed, he had told them that he was assaulted on his way home, and had been beaten and kicked several times to the head. There was no reported loss of consciousness. His only complaint before he went to bed was of a mild headache. Five hours later a family member called for an ambulance, as he was not responding adequately to speech or other stimuli.

**Fig. 1:**
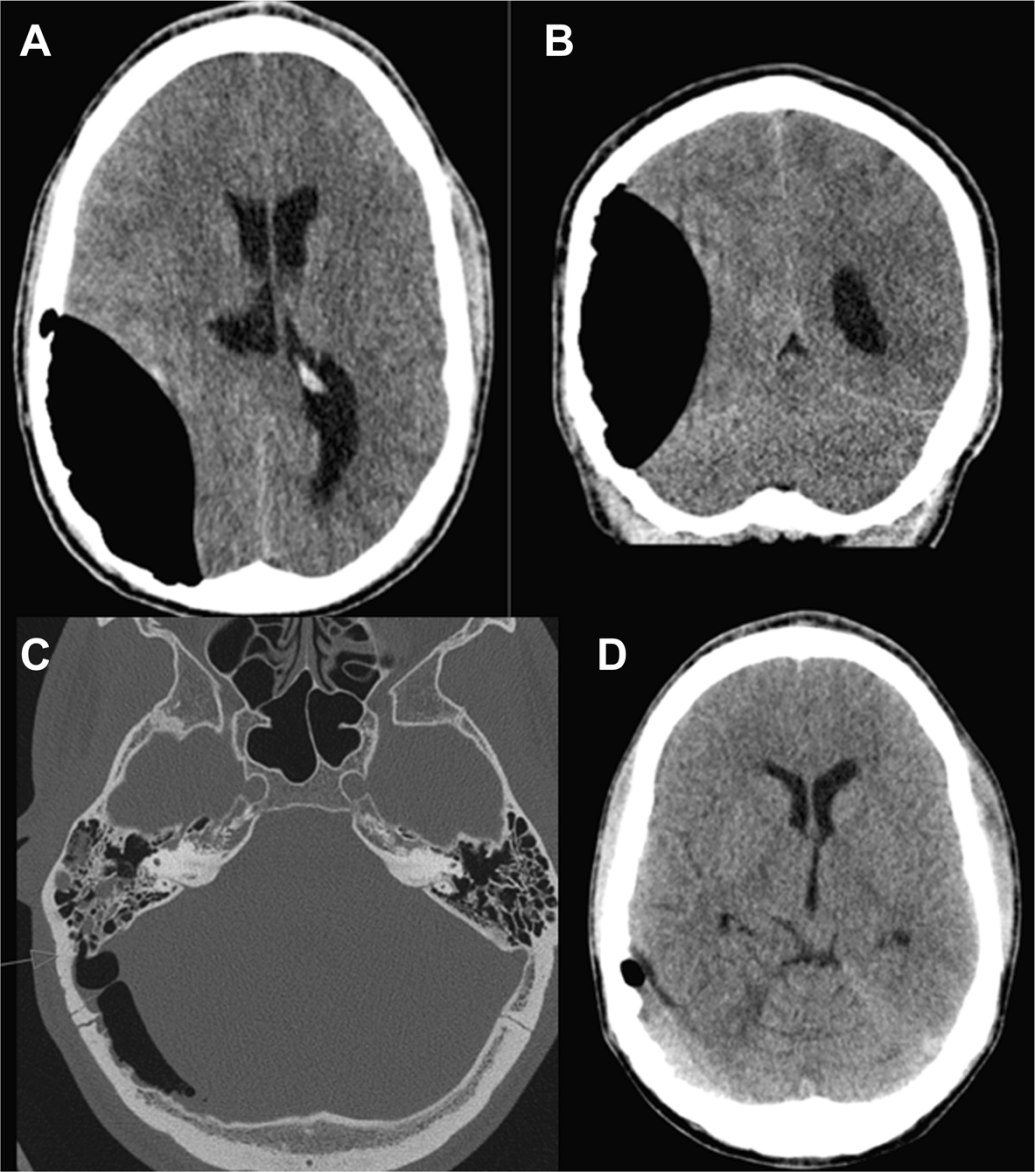
Imaging with computed tomography of the head revealed a 10cm×4cm expansive epidural lesion in the right temporoparietal region causing significant anteromedial compression of the right lateral ventricle and a midline shift of 7mm (**a** and **b**). A communication between the right mastoid sinus and the epidural space caused by a fracture was suspected (**c**). Postoperative control computed tomography the first postoperative day showed a small epidural bleed in the compartment that previously was filled with air, but the majority of the epidural air was removed and the midline was normalized (**d**)

In the emergency department he had a Glasgow Coma Scale (GCS) [4] score of 8 and anisocoria with a slight right-sided pupillary dilatation. He had bruises on his face and thorax, as well as a periorbital swelling. There were no signs of cerebrospinal fluid (CSF) rhinorrhoea or otorrhoea. He was otherwise clinically stable with normal vital parameters (blood pressure 125/60mmHg, regular heart rate/pulse 75/minute, oxygen saturation 100 %). The initial examination performed by a trauma team included X-ray imaging of his thorax and pelvis, that were found normal, as well as ultrasound of his abdomen, which showed no sign of free fluid. Imaging with computed tomography (CT) of his head revealed a 10cm×4cm expansive epidural lesion in the right temporoparietal region causing significant anteromedial compression of the right lateral ventricle and a midline shift of 7mm (Fig. 1a and b). The lesion had the attenuation values of air (−1000 Hounsfield units), and there were no signs of intracranial bleeding. There were no signs of herniation. CT also demonstrated a communication between the right mastoid sinus and the epidural space, and a fracture was suspected (Fig. 1c). He was immediately intubated and transported by helicopter at sea level to the nearest neurosurgical department, located approximately 180km away, where an emergency burr hole evacuation was performed to equalize the pressure. He experienced immediate recovery and was extubated the same day. In the evening he had a full GCS score of 15. On examination there were no neurological deficits. A postoperative control CT the first postoperative day showed a small epidural bleed in the compartment that previously was filled with air, but the majority of the epidural air was removed and the midline was normalized (Fig. 1d).

The following days he deteriorated and consecutive CT scans demonstrated increasing air entrapment. An indication for more invasive surgery was found, and a craniotomy with cranialization and sealing of the mastoid sinus with autologous musculature, an absorbable fibrin patch (Tachosil®) and fibrin glue (Tisseel®) was performed, to prevent further air entrapment. The dura was intact, and there was no leakage of cerebrospinal fluid. During the procedure several bony defects were found in his mastoid bone, and a mucosal membrane arising from the mastoid sinus found adherent to his skull and dura mater (that is, a mucocele) was removed.

He recovered quickly from this procedure, and postoperative CT scans were satisfactory, with re-expansion of the dura, resolution of the midline shift, and only minor residuals of the epidural air. He was discharged from the hospital 15 days after admission.

Unfortunately, 4 weeks later he deteriorated again, and complained of increasing headaches and diplopia. Renewed CT scans revealed increasing entrapment of the residual air, and a second surgery was performed. The same procedure was repeated, and a recanalization of the defect in the bone was found. Again, thorough sealing was obtained with bone wax, a fibrin patch and fibrin glue, and this time it ultimately turned out to be successful. The latest follow-up CT scan performed 4 months later was still found to be satisfactory.

## Discussion

Intracranial pneumocephalus was first described in an autopsy report of a trauma patient in 1866 [5]. Some years later Chiari reported a similar finding in an autopsy of a patient with chronic ethmoid sinusitis [6]. The usefulness of X-ray in diagnosing intracranial air was demonstrated by Luckett in 1913 [7], and 1 year later Wolff was the first to use the term *pneumocephalus* [8].

A previous review of the underlying causes indicated that trauma was the most common cause of pneumocephalus, associated with nearly 75 % of the cases, whereas neoplasm and infection were found in 13 % and 9 % of the cases, respectively [3]. In the same study, spontaneous or idiopathic pneumocephalus was only found in 0.6 % of the cases. Spontaneous otogenic pneumocephalus is considered very rare, and has been reported less than a dozen times in the literature [2].

Pneumocephalus is most easily diagnosed on CT [9], which can detect quantities of air as low as 0.5ml. Air appears dark black (that is, darker than CSF) with attenuation values of −1000 Hounsfield units [10], and will have a different distribution pattern depending on the localization. Depending on the underlying cause, the intracranial air can be distributed in the epidural space, subdural space or subarachnoid space, intraventricular or intracerebral, or a combination of these. A typical finding after burr holes or craniotomies is frontal air entrapment with a bilateral distribution, and has been described as the Mount Fuji sign [11], as it resembles the silhouette of the famous Japanese volcano.

The treatment depends on the underlying cause of air entrapment, but in most cases it resolves spontaneously with conservative care. In symptomatic postoperative pneumocephalus, treatment with 100 % oxygen has been shown to increase the rate of reabsorption [12]. Tension pneumocephalus producing significant symptoms is considered a neurosurgical emergency, and must be evacuated similar to an intracranial hematoma. Treatment options include puncture with a needle if there is an existing burr hole, or placement of a new burr hole. Otogenic pneumocephalus is also usually managed surgically, at least in the few cases reported in the literature, in an attempt to equalize pressure and close the fistula causing intracranial air entrapment. Due to its rarity, the optimal treatment is unknown. Closing of the fistula can be obtained with autologous muscle grafts, fascia flaps, bone wax, artificial fibrin patches or fibrin glue, most often in combination [2].

In our case we present a patient with a large, temporoparietal pneumocephalus, where air was introduced to the intracranial epidural space through a small defect in the mastoid bone, with a fistula into a mucous compartment. Due to the history of a significant blunt head trauma a few hours prior to admission, it was considered to be post-traumatic. As he experienced rapid clinical deterioration and life-threatening symptoms of herniation, immediate pressure relief was indicated, and obtained with a burr hole evacuation of the entrapped air. Although the patient experienced a short relief of symptoms caused by tension, a more invasive procedure was needed to resolve the condition. A craniotomy with cranialization of the mastoid sinus and thorough sealing of the defect in the mastoid bone was performed to prevent air from escaping through the sinus into the epidural space. Due to inadequate sealing, repeat surgery was needed 4 weeks later to eventually treat the problem. Based on the intraoperative findings of an extensive mucous membrane, it is reasonable to believe that a pre-existing mucocele originating from the mastoid sinus must have been a contributing factor, allowing air to enter the epidural space. In addition, a thinning of the skull bone adjacent to the epidural air suggests that there has been a mass lesion there for a substantial time. A possible mechanism could also be the rupture of this mucocele, most likely caused by the head trauma, allowing air to enter the epidural space from the mastoid sinus and causing tension pneumocephalus.

## Conclusions

Epidural tension pneumocephalus is a rare and potentially life-threatening condition, but treatable with the right management. To the best of our knowledge, a post-traumatic tension pneumocephalus caused by a pre-existing mucocele has not been reported in the literature. Unfortunately, we do not have any prior imaging of this patient to ultimately prove this theory.

## Consent

Written informed consent was obtained from the patient for publication of this case report and accompanying images. A copy of the written consent is available for review by the Editor-in-Chief of this journal.

## Abbreviations

CSF: Cerebrospinal fluid
CT: Computed tomography
GCS: Glasgow Coma Scale
